# Hair Dyes

**Published:** 1872-08

**Authors:** 


### HAIR DYES

Are almost all of them poisonous; medical publications abound in narrations, showing that they induce convulsions and various forms of obscure diseases, which in some instances have resisted all treatment, leaving the victim to suffer on without hope of relief; and as it is impossible to know what ingredients are contained in any article prepared for sale, and as the most efficient contain lead in its most poisonous forms, it is better either to let nature alone, or if any efforts are made to restore the natural color, use such mixtures as you can prepare for yourself. In some cases the following preparation will turn the hair black, if persevered in. The use of it is advised before trying those whose ingredients are not known.

Take equal parts by measure of bay rum, olive oil, and good brandy; shake it well on using, and wash the hair well with it, every morning.

